# Antitumor Activity of Luteolin Against Ehrlich Solid Carcinoma in Rats via Blocking Wnt/β-Catenin/SMAD4 Pathway

**DOI:** 10.7759/cureus.39789

**Published:** 2023-05-31

**Authors:** Heba Aljohani, Ahmed E Khodier, Mohammed M Al-Gayyar

**Affiliations:** 1 PharmD Program, University of Tabuk, Tabuk, SAU; 2 Pharmacology, Horus University, Faculty of Pharmacy, New Damietta, EGY; 3 Biochemistry, Mansoura University Faculty of Pharmacy, Mansoura, EGY; 4 Pharmaceutical Chemistry, University of Tabuk Faculty of Pharmacy, Tabuk, SAU

**Keywords:** wnt, smad4, luteolin, e-cadherin, β-catenin, ehrlich solid carcinoma (esc)

## Abstract

Background

Ehrlich solid carcinoma (ESC) is characterized by rapid proliferation and a short survival time. Because Ehrlich ascites carcinoma (EAC) resembles human cancer cells, Ehrlich solid and ascetic forms are commonly used to determine the anticancer effects of various compounds. Luteolin is a flavonoid compound found in many dietary sources, including carrots, peppers, celery, olive oil, peppermint, and oregano. Luteolin has potent anti-inflammatory, antidiabetic, antitumor, and antiapoptotic activities.

Aims

This study aims to investigate the antitumor activity of luteolin against ESC in rats by affecting the Wnt/β-catenin/SMAD4 pathway.

Methods

We introduced 0.15 ml of Ehrlich cells (2 × 10^6^) ESC into the left hind thighs of rats. After eight days of inoculation, the rats orally received 25 mg/kg of luteolin daily. We stained sections of tumor tissues with Masson’s trichrome. We used another part of the tumor tissue to assess gene and protein expression of Wnt, β-catenin, E-cadherin, and SMAD4.

Results

Treatment of carcinoma rats with luteolin increased the mean survival time and reduced tumor volume and weight. In addition, examination of tumor tissue stained with Masson’s trichrome showed loosely to densely packed collagen fibers in between neoplastic cells and scattered papillary expansion of a loose blue band of collagen expression along the covering adipose connective tissue and extending in a fine strand in between muscle fibers, which was ameliorated by treating rats with luteolin. Finally, treating ESC in rats with luteolin overexpressed E-cadherin and downregulated Wnt, β-catenin, and SMAD4.

Conclusions

We found luteolin has antineoplastic activity against ESC by reducing tumor size and weight while improving the structure of muscle cells. It works by suppressing Wnt, β-catenin, and SMAD4, resulting in decreased tumor cell proliferation and differentiation. Additionally, luteolin overexpresses E-cadherin, leading to reduced tumor cell invasion and metastasis.

## Introduction

A tumor is a pathologic condition characterized by an abnormally high and unnatural rate of cell division. Solid tumors make up around 85% of all human malignancies. It is the second-leading cause of death [1]. The Global Cancer Statistics 2020 report indicated 19.3 million newly diagnosed cancer patients and 10 million cancer-related deaths [2]. Despite the considerable advancements in anticancer technology, no widespread treatment for malignant diseases exists. The foundation of conventional chemotherapy is to kill cancer cells without affecting adjacent normal cells. However, in practice, cytotoxic drugs have little specificity, leading to systemic toxicity and severe side effects such as hair loss and organ damage. Therefore, there is always an increasing necessity for evaluating new active anticancer drugs. The Ehrlich tumor is derived from spontaneous murine mammary adenocarcinoma and transforms into an ascites form through intraperitoneal serial passages. This Ehrlich ascites model is commonly used in experimental cancer studies due to its effectiveness in producing neoplastic cells and accuracy in predicting survival time. The solid form of Ehrlich carcinoma (ESC) is developed through subcutaneous inoculation and is a valuable tool for researching antineoplastic drugs [3].

Numerous phytochemicals offer the possibility of brand-new anticancer compounds. One of these compounds is luteolin (3',4',5,7-tetrahydroxyflavone), a flavonoid abundant in many plants and used to treat various diseases such as inflammation and hypertension. The anticancer activity of luteolin is attributed to the prevention of cell growth, metastasis, and angiogenesis [4]. Studies have reported that it produces therapeutic effects against ESC via activation of apoptosis [5] and enhancement of expression of p53 and cyclin D1 [6]. However, no previous study illustrated the anticancer effect in ESC via affecting the Wnt/β-catenin pathway. The aim of our study is to explore the potential antitumor effects of luteolin against Ehrlich ascites carcinoma (EAC) in rats through its impact on the Wnt/β-catenin/SMAD4 pathway.

## Materials and methods

Animals and treatment outlines

We used 30 Sprague-Dawley rats, weighing 180-200 g, under standard temperature conditions and a regular 12-hour light and dark cycle. The Research Ethics Committee of Horus University’s Faculty of Pharmacy approved the working protocol under number P2023-003. We divided the rats into three groups. The control group contained ten rats kept without treatment for the whole experiment period. The ESC group had ten rats subjected to an intramuscular injection of 0.15 ml of Ehrlich cells (2 × 106) in the thigh of the left hind leg. In the ESC treated with luteolin, after induction of ESC in ten rats and after a solid tumor had appeared on day 8, which is the time taken for the detectable mass of tumor to appear based on the amount and viability of the injected cells, the rats were given 25 mg/kg of luteolin (Sigma-Aldrich Chemical Company, Burlington, MA) by oral gavage on day zero. We treated the rats with garcinol for three weeks.

Sample collection 

We separated the whole tumor area from the thigh of the left hind leg and then measured and weighed it. Part of the muscle tissue was fixed in formalin and subsequently used for morphologic investigation. Another part was homogenized in sodium-potassium phosphate buffer and centrifuged for the isolation of the supernatant, which was stored at −80 °C.

Staining muscle sections with Masson trichrome 

Muscle tissues were cut into 5-µm sections and stained with Masson’s trichrome. The fibrotic scores were calculated by examining ten fields stained by hematoxylin and eosin in each sample at high power.

Enzyme-linked immunosorbent (ELISA) assay 

Following the manufacturer’s instructions, we used commercially available ELISA kits to assess β-catenin, cadherin, and SMAD4 (MyBioSource, Inc., San Diego, California, USA).

Quantitative real-time polymerase chain reaction (RT-PCR)

The gene expression of Wnt, β-catenin, cadherin, SMAD4, and GAPDH mRNA levels in rat muscle lysate was performed as described previously by our group [7-9]. Table 1 shows the gene-specific PCR primers we used.

**Table 1:** Primer sets used to detect gene expression in rats.

Name	Sequence		Reference sequence
GAPDH	Forward	5`-CCATCAACGACCCCTTCATT-3`	NM_017008.4
	Reverse	5`-CACGACATACTCAGCACCAGC-3`	
Wnt	Forward	5`-CTGACCTGATGCAGACGCAAG-3`	NM_003391
	Reverse	5`-AGGAGCCACCTGTAGCTCTCATGTA-3`	
β-catenin	Forward	5`-TCCGTCGCCGGTCCACACCC-3`	NM_031144.3
	Reverse	5`-TCACCAACTGGGACGATATG-3`	
E-Cadherin	Forward	5`-ATCCTGGCCCTCCTGATTCT-3`	NM_031334.1
	Reverse	5`-CGGGTATCGTCATCTGGTGG-3`	
SMAD4	Forward	5`-GGATGAAGTCCTGCACACCA-3`	NM_019275.3
	Reverse	5`-GTTGAAGCACTGCCACCTTG-3`	

Statistical analysis

For the presentation of quantitative variables, mean ± standard error was used. A one-way analysis of variance (ANOVA) was used for comparison between groups, followed by a post-hoc Bonferroni correction test. We conducted statistical analyses using IBM SPSS Statistics, version 20 (Chicago, Illinois, USA). We predefined statistical significance as P < 0.05.

## Results

Antitumor activity of luteolin in ESC

**Figure 1:**
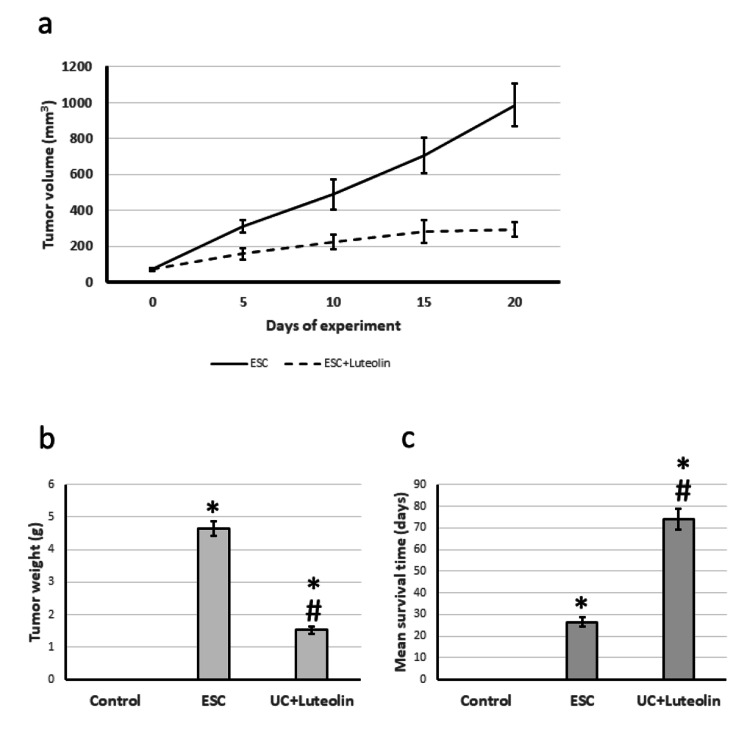
Effect of ESC and 25 mg/kg luteolin on tumor volume over experiment period (a), tumor weight at the last day of the experiment (b), and rats mean survival time (c). *Significant difference against control group at p<0.05. # significant difference against ESC group at p<0.05. ESC: Ehrlich solid carcinoma.

We observed an increased volume of tumors over the days of the experiment, associated with an increase in the weight of cancer on the day of sacrifice. Treatment of the ESC group with luteolin significantly reduced the tumor volume and weight compared to the control group. In addition, treating ESC rats with luteolin significantly increased the mean survival time from 28 to 74 days (Figure 1).

Effect of luteolin on ESC-induced alteration in cells morphology

**Figure 2:**
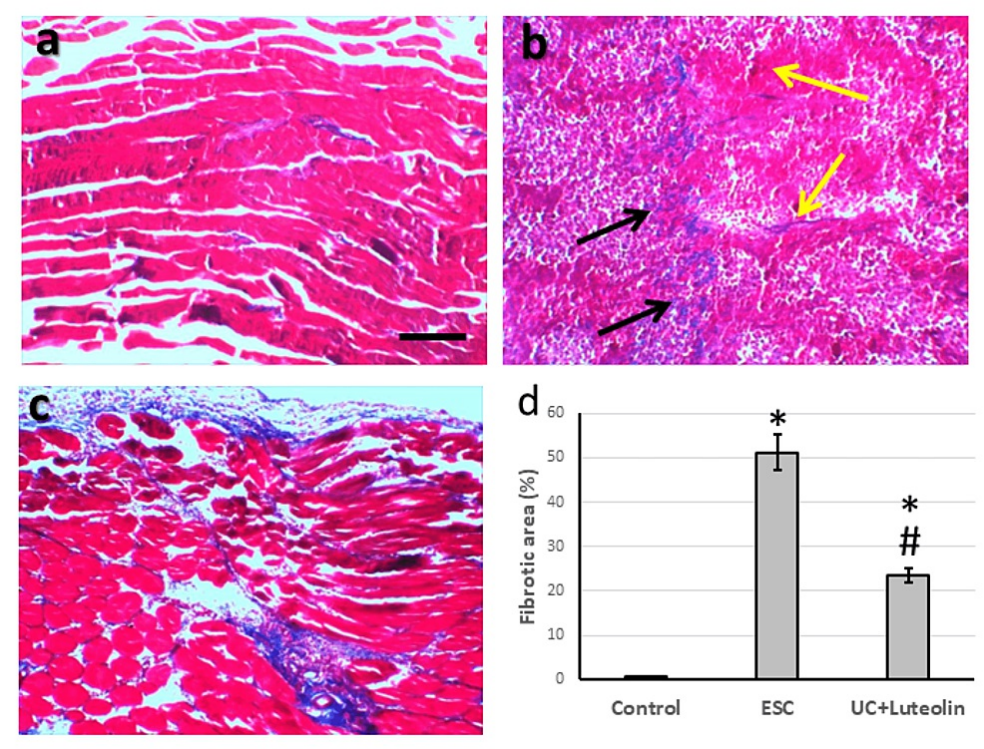
Muscle sections stained with Masson trichrome in control group (a), Ehrlich solid carcinoma (ESC, b) and ESC treated with 25 mg/kg luteolin (c). Fibrotic area was determined in 10 fields of high field power and expressed as mean ± standard deviation (d). Black arrow represented loosely to densely packed collagen fibers in between neoplastic cells. Yellow arrows represented scattered papillary expansion of loosely blue band of collagen expression along the covering adipose connective tissue and extending in a fine strands in between muscle fibers. *Significant difference as compared with the control group at p<0.05. ^#^Significant difference as compared with ESC group at p<0.05. Scale bar 100 μm.

Representative microimages from the control group stained with Masson’s trichrome showed a typical appearance of muscle tissue. The microimages of ESC showed loosely to densely packed collagen fibers between neoplastic cells (black arrows). They scattered along the papillary expansion of a loose blue band of collagen expression along the covering adipose connective tissue and extended in a fine strand in between muscle fibers (yellow arrows). Finally, treatment of ESC with luteolin greatly improved muscle fiber. In parallel, as compared with the ESC group, luteolin significantly decreased the fibrotic score (Figure 2).

Effect of luteolin on ESC-induced expression of Wnt/β-catenin 

**Figure 3:**
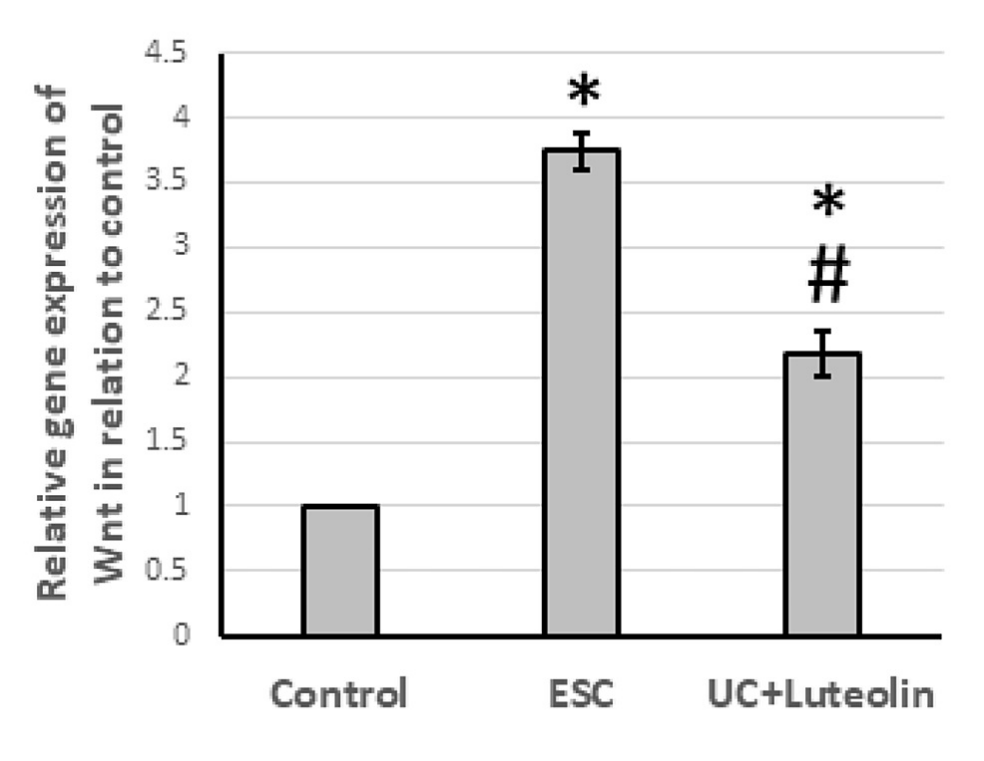
Effect of ESC and 25 mg/kg luteolin on gene expression of Wnt. *Significant difference as compared with control group at p<0.05. ^#^Significant difference as compared with ESC group at p<0.05. ESC: Ehrlich solid carcinoma.

**Figure 4:**
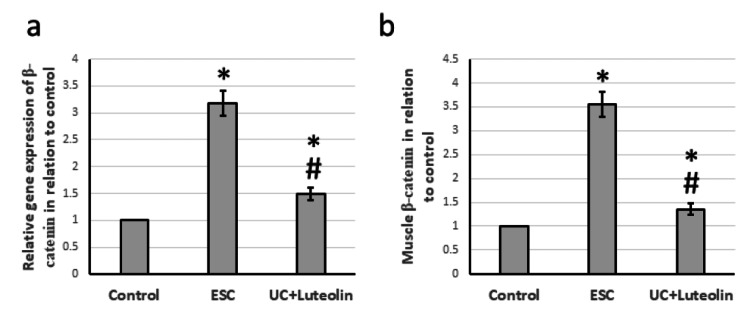
Effect of ESC and 25 mg/kg luteolin β-catenin gene expression (a) and protein level (b). *Significant difference as compared with control group at p<0.05. ^#^Significant difference as compared with ESC group at p<0.05. ESC: Ehrlich solid carcinoma.

After ESC, there was a notable increase in the gene expression of both Wnt and β-catenin, by 3.74- to 3.18-fold, respectively. This was accompanied by a 3.56-fold increase in the levels of β-catenin in the muscle compared to the control group. However, treatment with luteolin in rats demonstrated a reversal of these effects, as shown in Figures 3-4.

Effect of luteolin on ESC-induced reduction of the expression of E-cadherin 

**Figure 5:**
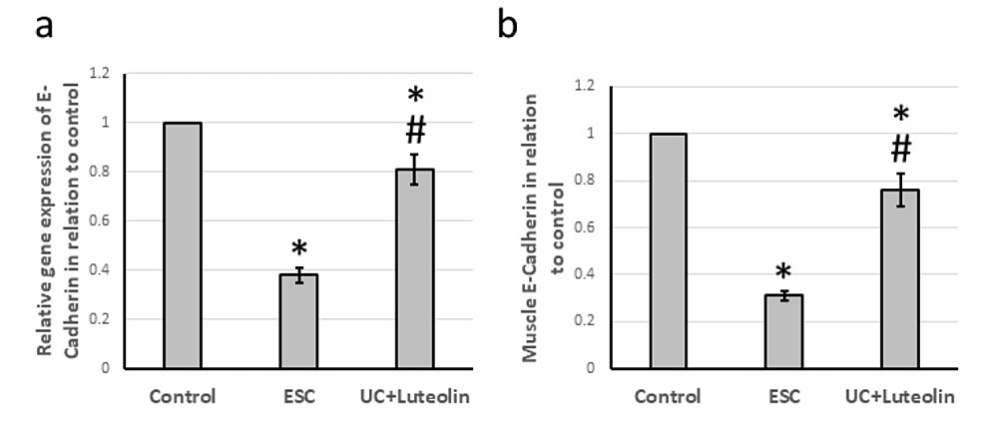
Effect of ESC and 25 mg/kg luteolin on E-cadherin gene expression (a) and protein level (b). *Significant difference as compared with control group at p<0.05. ^#^Significant difference as compared with ESC group at p<0.05. ESC: Ehrlich solid carcinoma.

ESC caused a 62% reduction in the gene expression of E-cadherin, associated with a 69% decrease in the muscle E-cadherin level compared to that of the control group. Treatment of ESC rats with luteolin significantly increased the expression of E-cadherin, but it was still lower than that of the control group (Figure 5).

Effect of luteolin on ESC-induced activation of SMAD4 

**Figure 6:**
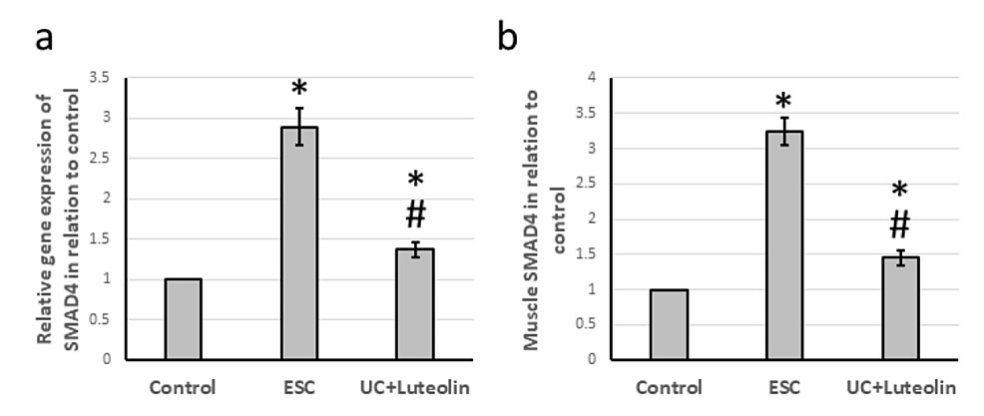
Effect of ESC and 25 mg/kg luteolin on gene expression of SMAD4 (a) and its muscle level (b). *Significant difference as compared with control group at p<0.05. ^#^Significant difference as compared with ESC group at p<0.05. ESC: Ehrlich solid carcinoma.

ESC resulted in a 2.89-fold elevation in the gene expression of SMAD4, associated with a 3.24-fold increase in muscle levels of SMAD4 as compared with the levels of the control rats. Treatment of ESC rats with luteolin significantly reduced the expression of SMAD4 compared to that of the ESC rats, but it was still higher than that of the control group (Figure 6).

## Discussion

ESC is a fast-growing carcinoma used as an in vivo experimental model for checking proposed antitumor compounds. Ehrlich cancer cells can develop ascites when implanted intraperitoneally and solid forms when implanted subcutaneously [10]. We implanted the Ehrlich cells subcutaneously inside the thigh of the rats’ left hind leg, which caused an elevation in the volume of the portion over the week of the experiment because of tumor growth. We confirmed the tumor growth after rat sacrifice by weighing the tumor after its separation from the leg. Moreover, we checked microimages stained with Masson’s trichrome that showed packed collagen fibers in between neoplastic cells and the expansion of a loose blue band of collagen expression along the covering adipose connective tissue and extending in a fine strand in between muscle fibers. Treating ESC rats with luteolin significantly reduced the tumor volume and weight associated with an elevation of the mean survival time of the rats from 28 to 74 days. Researchers previously reported that luteolin produces therapeutic effects against ESC via activation of apoptosis [5] and enhancement of expression of p53 and cyclin D1 [6]. However, no previous study illustrated the anticancer effect in ESC via affecting the Wnt/β-catenin pathway.

The Wnt signaling pathway plays a significant role in the development of cancer. The upregulation of oncogenes and the maintenance of cancer cells occur because of the expression of the Wnt pathway. Thus, targeting the Wnt signaling pathway therapeutically can prevent tumor growth in cancers such as hepatocellular carcinoma [11], prostate cancer [12], and colorectal cancer [13]. β-catenin, a complex protein, also plays a critical role in tumor development, growth, regeneration, and invasion by promoting cell-to-cell adhesion and activating extracellular matrix components [14]. Its activation leads to the overexpression of downstream target genes, resulting in carcinogenesis [11]. SMAD4, a downstream of β-catenin, is overexpressed in various types of cancer, and deleting the SMAD4 gene has protective effects against pancreatic cancer [15]. Our research found that ESC increased the expression of Wnt and β-catenin and reduced SMAD4 levels. Treating ESC rats with luteolin significantly improved these effects.

E-cadherin is a calcium-dependent transmembrane glycoprotein that serves as an adhesion molecule belonging to the superfamily of cadherins. It also provides homotypic cell-to-cell interplay [16]. E-cadherin’s cytoplasmic tail contains a β-catenin binding domain attached to β-catenin, forming a complex that helps epithelial cell integrity and cell-to-cell adhesion [17]. It is a well-known tumor suppressor because it blocks the release of cells from the tumor site, preventing their migration and metastasis [18]. Therefore, deactivation or downregulation of E-cadherin enhances the tumor’s metastatic potential. However, we found a significant reduction in both gene and protein expression of E-cadherin in ESC rats, which we reversed by treating rats with luteolin. In contrast, researchers previously reported that luteolin induces the expression of E-cadherin, leading to the inhibition of invasion of prostate cancer P3 cells [19].

**Figure 7:**
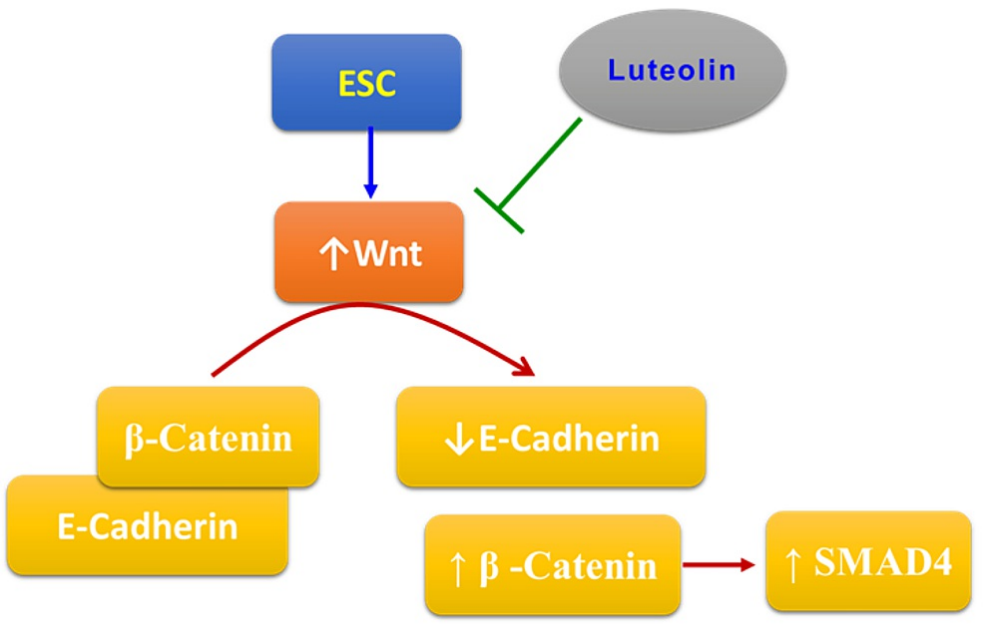
The mechanism of the protective effects of luteolin in ESC. ESC: Ehrlich solid carcinoma. Image Credits: The authors of the manuscript.

Luteolin represents a new and safe anticancer compound that is highly available and cheap. However, more experiments are needed to approve its anticancer effects and discover the whole mechanism of action. Figure 7 summarizes the mechanism of action of luteolin in ESC. Although the current study provided evidence of the ability of luteolin to produce therapeutic effects against ESC, it does have some limitations that are represented by different metabolic pathways in rats than those in humans, leading to different effects of some drugs. Moreover, we used ELISA and PCR techniques to elucidate the expression of the Wnt/β-catenin/SMAD4 and E-cadherin pathways with some limitations, such as low amplification yield and high background.

## Conclusions

We conducted the current study to discover the potential chemotherapeutic activity of luteolin against ESC. Because luteolin reduced the tumor size and weight associated with the improvement of the structure of muscle cells, we approved its use to produce antineoplastic activity against ESC. Upon investigation of the mechanism of the therapeutic effects of luteolin, we found that luteolin reduced tumor cell proliferation and differentiation, as indicated by the suppression of Wnt, β-catenin, and SMAD4. In addition, as indicated by the overexpression of E-cadherin, luteolin reduced tumor cell invasion and metastasis.
